# Vegetarian, pescatarian and flexitarian diets: sociodemographic determinants and association with cardiovascular risk factors in a Swiss urban population

**DOI:** 10.1017/S0007114520001762

**Published:** 2020-10-28

**Authors:** Hannah Wozniak, Christophe Larpin, Carlos de Mestral, Idris Guessous, Jean-Luc Reny, Silvia Stringhini

**Affiliations:** 1 Department of General Internal Medicine, Geneva University Hospitals, 1205 Geneva, Switzerland; 2 Unit of Population Epidemiology, Department of Primary Care Medicine, Geneva University Hospitals, 1205 Geneva, Switzerland; 3 University Centre for General Medicine and Public Health, University of Lausanne, 1011 Lausanne, Switzerland

**Keywords:** Vegetarian diets, Pescatarian diet, Flexitarian diet, Vegetarian prevalence, Cardiovascular risk factors, Sociodemographic factors, Diet trends

## Abstract

Prevalence and trends of different vegetarian diets remain unknown, with estimates varying depending on the source. Evidence suggests that vegetarian diets are associated with a more favourable cardiovascular risk profile. The present study aimed to assess the prevalence and trends of different types of vegetarian diets in a population-based representative sample, sociodemographic characteristics of participants following such diets and the association of these diets with cardiovascular risk factors. Using repeated cross-sectional population-based surveys conducted in Geneva, Switzerland, 10 797 individuals participated in the study between 2005 and 2017. Participants were classified as vegetarians, pescatarians, flexitarians or omnivores using an FFQ. Sociodemographic and cardiovascular risk factors were evaluated through questionnaires, anthropometric measurements and blood tests. Findings show prevalence of vegetarians increased from 0·5 to 1·2 %, pescatarians from 0·3 to 1·1 % and flexitarians remained stable at 15·6 % of the population over the study period. Compared with omnivores, vegetarians were more likely to be young (OR 2·38; 95 % CI 1·01, 5·6), have higher education (OR 1·59; 95 % CI 1·01, 2·49) and lower income (OR 1·83; 95 % CI 1·04, 3·21); pescatarians and flexitarians were more likely to be women (pescatarian: OR 1·81; 95 % CI 1·10, 3·00; vegetarian: OR 1·57; 95 % CI 1·41, 1·75) and flexitarians were also more likely to have a lower income (OR 1·31; 95 % CI 1·13, 1·53). Participants who adhered to any diet excluding/reducing meat intake had lower BMI, total cholesterol and hypertension compared with omnivores. The present study shows an increase in the prevalence of vegetarians over a 13-year period and suggests that the different vegetarian diets assessed are associated with a better cardiovascular risk profile.

In Western countries, it is increasingly popular to consciously reduce the consumption of meat, mainly due to environmental, ethical and health concerns^(1,2)^. However, the proportion of the population following vegetarian or flexitarian diets remains unknown as estimates vary widely depending on the source^(2)^. When estimates are driven by online or telephone surveys sponsored by vegetarian associations, the prevalence of self-reported vegetarians can be up to 10–14 %^(2–5)^. However, in population-based studies, national surveys, or independent analysis, only 1–4 % of the population reports following a vegetarian diet^(6–14)^.

An accurate estimation of the prevalence of individuals following a vegetarian diet is also complicated by the fact that a large number of different types of dietary regimens fall under the definition of ‘vegetarian’. Individuals may define themselves as vegetarians depending on the questions that are being asked or on the definition adopted in the specific survey. Indeed, different types of vegetarian diets can be identified. The strictest regimen is the vegan diet, where all animal products are avoided. Lacto-ovo vegetarians, simplified as vegetarians, include in their diet animal-derived products such as eggs and dairy products, but exclude meat or fish. Pescatarians are defined as vegetarians who also consume fish and seafood products^(1,15)^. More recently, the flexitarian diet (or semi-vegetarian) has been gaining popularity and identifies individuals eating meat occasionally, with no clear consensus on quantities of meat consumed per week or month required for falling under this category^(16)^. Omnivores are defined as eating indifferently animal and plant products.

In the current literature, it is difficult to find reliable data on the prevalence of vegetarians in the general population, and there is even less data on trends in the prevalence of these diets. Furthermore, few studies have attempted to characterise individuals following a vegetarian diet. Most of these reported that individuals following a vegetarian diet tend to be women, younger, smoke less and have a higher education^(9,17)^. Nonetheless, there seems to be contradictory evidence about the link between income and following a vegetarian diet^(9,18)^.

The medical community is showing growing interest in the potentially beneficial effects of a vegetarian diet, as shown by the increasing number of studies on the subject^(19–23)^. Cardiovascular risk factors such as elevated BMI, hypertension, hypercholesterolaemia and diabetes seem to be less prevalent in the population following different vegetarian diets^(22,24–26)^. Furthermore, some meta-analyses have shown a link between vegetarian diet and a protective effect on IHD and certain cancers^(21)^. Finally, some but not all evidence suggests a link between following a vegetarian diet and reduced all-cause mortality^(27,28)^.

The main purpose of the present study is to reliably assess the prevalence and temporal trends of different types of vegetarian diets in a yearly Swiss cross-sectional study. The secondary objectives are to analyse the sociodemographic characteristics of the cohort and to assess the possible correlation between the different types of vegetarian diets and cardiovascular risk factors. With the presumed health benefits of different vegetarian diets, it is essential to better identify and characterise who in the general population is following such diets.

## Materials and methods

Data were collected using the *Bus Santé* study, a cross-sectional population-based study in the State of Geneva, Switzerland conducted annually since 1993^(29,30)^. The aim is to collect sociodemographic data and information about cardiovascular risk factors from a representative sample of the Swiss adult population. Each year, about 1000 participants aged from 18 to 74 years are selected to participate in the study and individuals cannot be included more than once in the study. Eligible participants are identified from an annual residential list made by the Government of Geneva. Potential participants are contacted first by mail. If no answer is given, eligible participants can receive up to seven phone calls and up to two more mails. People who do not answer are replaced by another eligible participant. People who refuse to participate are not replaced. Participants are met either at the Geneva University Hospital or at a mobile medical unit (a bus), facilitating access to all suburban areas. Participation rate varied between 45 and 55 % with yearly variations.

First, participants receive a questionnaire at home about lifestyle habits (smoking, physical activity and nutrition), sociodemographic information and health, including questions regarding cardiovascular risks factors. The dietary habits are assessed using an FFQ with a recall period of 4 weeks. Secondly, participants undergo a face-to-face interview with trained nurses who verify the completeness of the questionnaires and take anthropometric measures including height and weight, according to standard procedures. For weight measurement, the participant is weighed on a medical scale and has to take off his shoes and be lightly dressed. Height is measured using a medical gauge. Blood pressure is measured three times after 10 min of rest in a sitting position with a cuff size adjusted to the arm circumference. The average of the three blood pressure measures is used for the analyses. A blood test is performed including the measurement of fasting plasma glucose, total cholesterol and TAG.

The *Bus Santé* study complied with the declaration of Helsinki and is approved by the institutional ethical committee of the University of Geneva. Furthermore, patients gave their written consent. No financial compensation was given to participants.

### Food frequency questionnaire

The FFQ used in the present study has been validated in the Geneva population^(31)^ and has been used in the previous studies^(32,33)^. It is based on ninety-seven different food items.

For each item, the participant must indicate the size of the portion consumed (smaller, equal or larger than a reference size) and the frequency at which it was consumed over the last 4 weeks (<1× over the last 4 weeks up to twice a day)^(34)^.

### Type of diet

Participants were classified according to the type of diet based on the FFQ results. Participants were classified as omnivores when they ate meat >1/week; as vegetarians when they excluded red meat, poultry and fish from their diet but ate dairy products and eggs; as pescatarians when they consumed fish, in addition to dairy products and eggs but did not eat red meat or poultry^(15)^ and as flexitarians when they included eggs and dairy products in their daily diet and red meat or poultry at a frequency of ≥1/month but ≤1/week^(19,28,35)^.

### Cardiovascular risk factors

We chose to focus on five cardiovascular risk factors: overweight, hypertension hypercholesterolaemia, diabetes and smoking.

BMI was calculated as weight/height^2^. Participants were classified as having normal BMI (BMI < 25 kg/m^2^), as being overweight (BMI ≥ 25 and <30 kg/m^2^) or as being obese (BMI ≥ 30 kg/m^2^). Hypertension was defined as either one measurement of the systolic blood pressure ≥140 mmHg or mean blood pressure ≥90 mmHg or if medications against high blood pressure were taken or as having a previous diagnosis of hypertension. Hypercholesterolaemia was defined as having total blood cholesterol > 6·5 mmol/l and HDL < 1 mmol/l or if medications against hypercholesterolaemia were taken or as having a previous diagnosis. Diabetes was defined as a fasting glucose > 7 mmol/l or if medications against diabetes were taken or as having a previous diagnosis. Smoking was defined as being a current smoker.

Total cholesterol, systolic and diastolic blood pressure, fasting plasma glucose and BMI were also assessed as continuous biomarkers in relation to the different types of diet.

### Statistical analysis

The analytical sample comprised all the participants included in the Bus Santé study from 2005 to 2017. Although the *Bus Santé* study started in 1993, biomarkers of interest for our study became available from 2005 onwards, which is why earlier data were not included in the analysis. We used *χ*^2^ test for differences in dietary pattern between men and women in binary/categorical variables and student *t* test in continuous outcomes. All assumptions were met for normality and homoscedasticity. Given the sex differences in diet patterns, we conducted analyses separately for men and women, as well as in the overall sample. To calculate the prevalence and 95 % CI, we applied margins after logistic regression, adjusting for age in sex-specific models and for age and sex in the overall model. To assess the change in prevalence between the first (%_start_) and last (%_last_) survey periods, we used the following formula: ((%_last_–%_start_)/(%_start_)) × 100. To test for linear trend in the prevalence of diet patterns, we included the survey period variable as continuous predictor in the model. To assess for trends in food groups (i.e. meat type consumption) and for the association between diet patterns and biomarkers, we applied multivariable linear regression. To investigate the association between diet patterns and sociodemographic and cardiovascular risk factors, we used multinomial logistic regression, adjusting the estimates for age, sex and survey period. We further adjusted the estimates for BMI in sensitivity analyses to assess its role in the link between diet type and cardiovascular risk factors. All statistical analyses were conducted using STATA version 15 (Stata Corp.). The level of significance was set to *P* < 0·05. Results for daily dietary intake are expressed as *β*-coefficients and 95 % CI, for biomarkers as means and standard deviations and for the association between diet patterns and sociodemographic and cardiovascular risk factors as OR and 95 % CI.

## Results

### Baseline characteristics and prevalence

A total of 10 797 participants were included in the analyses, which spanned over a period of 13 years, from 2005 until 2017 (Table 1). Of the total number of participants, 51 % were women and the mean age was 48·9 (sd 13·4) years. The mean BMI was 25·1 (sd 4·4) kg/m^2^, with 33·3 % of participants being overweight and 13·3 % being obese. Current smokers were 22 % of the sample. In the sample, 46·7 % had been through higher education or had a university degree.

**Table 1.** Description of sample, Bus Santé study 2005–2017 (*n* 10 797)* (Numbers and percentages; mean values and standard deviations)

	*n*	Total			Men			Women			
		*n*		%	*n*		%	*n*		%	*P*
Sample size (*n*)		10 797			5246			5551			
Age (years)	10 797										0·16
Mean			48·9			49·1			48·8		
sd			13·4			13·4			13·3		
Age categories	10 797										0·24
18–44		4450		41·2	2146		40·9	2304		41·5	
45–64		4758		44·1	2297		43·8	2461		44·3	
65+		1589		14·7	803		15·3	786		14·2	
Living alone	10 290	3596		35·0	1555		31·1	2041		38·5	<0·001
Swiss nationality	10 289	6827		66·4	3236		64·8	3591		67·8	0·001
University degree	10 148	4735		46·7	2408		49·2	2327		44·3	<0·001
Manual occupation	9772	2652		27·1	1436		29·9	1216		24·5	<0·001
Household income	9367										
<5000 CHF/month		2078		22·2	892		19·3	1186		25·0	<0·001
5000–9500 CHF/month		3668		39·2	1702		36·8	1966		41·5	<0·001
>9500 CHF/month		3621		38·6	2033		43·9	1588		33·5	<0·001
Dietary pattern	10 797										
Omnivorous		8965		83·0	4526		86·3	4439		80·0	<0·001
Flexitarian		1682		15·6	662		12·6	1020		18·4	<0·001
Pescatarian		66		0·6	24		0·5	42		0·8	0·02
Vegetarian		84		0·8	34		0·7	50		0·9	0·07
Current smoker	10 281	2264		22·0	1161		23·3	1103		20·8	0·003
BMI (kg/m^2^)											<0·001
Mean			25·1			26·0			24·3		
sd			4·4			3·9			4·7		
BMI categories	10 240										
Normal		5466		53·4	2203		43·4	3263		63·2	<0·001
Overweight		3414		33·3	2140		42·2	1274		24·7	<0·001
Obese		1360		13·3	732		14·4	628		12·2	<0·001
Hypercholesterolaemia	9438	3925		41·6	2093		47·1	1832		36·7	<0·001
Hypertension	10 041	2981		29·7	1683		34·5	1298		25·1	<0·001
Diabetes	9576	654		6·8	382		8·2	272		5·5	<0·001

CHF, Swiss Francs (1 CHF = 1·01 USD as of 14 November 2019).

* *P* value for difference between men and women from *t* test for continuous variables and from *χ*^2^ test for binary/categorical variables. Hypertension was defined as having a previous diagnosis or blood pressure ≥140/90 mmHg. Hypercholesterolaemia was defined as having a previous diagnosis or having total blood cholesterol >6·5 mmol/l and HDL <1 mmol/l. Diabetes was defined as self-reported diabetes or a fasting plasma glucose level of ≥7 mmol/l.

Within the studied population, there were eighty-four vegetarians (0·8 %), sixty-six pescatarians (0·6 %) and 1682 flexitarians (15·6 %). Due to the extremely low prevalence of vegans (<10 people over the 13 years of data collection), no sub-group analysis was conducted for this category.

### Trends for diets and meat consumption

Over the 13 year study period, the prevalence of a vegetarian diet increased from 0·5 to 1·2 % (*P* = 0·03; Table 2). In analysis stratified by sex, an increase in the prevalence of vegetarians was significant for women but not for men. The proportion of participants following a pescatarian diet also increased significantly from 0·3 to 1·1 % (*P* < 0·01) with a significant increase for men but not for women. The prevalence of flexitarians remained stable during the survey period (15·6 %). Over the 13 years, beef intake significantly decreased by 15 % for women and by 9 % for men, which was barely non-significant (*P* = 0·06), while poultry intake significantly increased by 8 and 10 %, respectively. Despite those variations, the overall total meat intake remained stable (Table 3). When adjusted for education, occupation and income, there was no longer a significant decrease in poultry intake (online Supplementary Table S1).

**Table 2.** Trends in the prevalence of diet patterns by sex, Bus Santé study 2005–2017 (*n* 10 797) (Numbers; percentage values and 95 % confidence intervals)*

	2005–2009 (*n* 2340)			2010–2011 (*n* 1959)			2012–2013 (*n* 2061)			2014–2015 (*n* 2182)			2016–2017 (*n* 2256)			Δ %	*P*_trend_
	*n*	%	95 % CI	*n*	%	95 % CI	*n*	%	95 % CI	*n*	%	95 % CI	*n*	%	95 % CI		
Total																	
Omnivorous	1947	83·2	81·7, 84·7	1650	84·2	82·6, 85·8	1737	84·3	82·7, 85·8	1808	82·9	81·3, 84·4	1824	80·9	79·2, 82·5	−3	0·02
Flexitarian	376	16·1	14·6, 17·6	288	14·7	13·1, 16·3	290	14·1	12·6, 15·6	347	15·9	14·4, 17·4	381	16·9	15·4, 18·4	5	0·15
Pescatarian	6	0·3	0·1, 0·5	11	0·6	0·2, 0·9	14	0·7	0·3, 1·0	10	0·5	0·2, 0·7	25	1·1	0·7, 1·5	327	<0·01
Vegetarian	11	0·5	0·2, 0·8	10	0·5	0·2, 0·8	20	1·0	0·6, 1·4	17	0·8	0·4, 1·2	26	1·2	0·7, 1·6	145	0·03
Men																	
Omnivorous	991	86·7	84·7, 88·7	828	86·5	84·4, 88·7	866	86·1	83·9, 88·2	904	85·7	83·6, 87·8	937	86·4	84·3, 88·4	0	0·37
Flexitarian	149	13·0	11·1, 15·0	120	12·5	10·4, 14·6	125	12·4	10·4, 14·5	138	13·1	11·1, 15·1	130	12·0	10·1, 13·9	−8	0·89
Pescatarian	3	0·3	0·0, 0·6	3	0·3	0·0, 0·7	6	0·6	0·1, 1·1	5	0·5	0·1, 0·9	10	0·9	0·4, 1·5	254	<0·01
Vegetarian	0	0·0		6	0·6	0·1, 1·1	9	0·9	0·3, 1·5	8	0·8	0·2, 1·3	8	0·7	0·2, 1·3	17	0·32
Women																	
Omnivorous	956	79·9	77·6, 82·1	822	82·0	79·7, 84·4	870	82·5	80·3, 84·8	904	80·2	77·9, 82·5	887	75·8	73·3, 78·2	−5	0·02
Flexitarian	227	19·0	16·7, 21·2	168	16·8	14·5, 19·1	165	15·7	13·5, 17·9	209	18·5	16·3, 20·8	251	21·4	19·1, 23·8	13	0·10
Pescatarian	6	0·5	0·1, 0·9	8	0·8	0·3, 1·4	8	0·8	0·2, 1·3	5	0·4	0·1, 0·8	15	1·3	0·6, 1·9	156	0·06
Vegetarian	8	0·7	0·2, 1·1	4	0·4	0·0, 0·8	11	1·0	0·4, 1·7	9	0·8	0·3, 1·3	18	1·5	0·8, 2·2	130	0·04

* Prevalence in percentage and 95 % CI from margins after logistic regression, adjusted for age in sex-specific models and for sex in age-specific models. *P* for linear trend across survey years. *N* represents the raw number of participants reporting each dietary type. Δ represents the percentage change in the prevalence of dietary pattern between 2005–2009 and 2016–2017, except for vegetarian diet pattern among men, for which the starting period is 2010–2011.

**Table 3.** Trends in the mean consumption of meat type by sex, Bus Santé study 2005–2017* (Mean values and 95 % confidence intervals)

	2005–2009		2010–2011		2012–2013		2014–2015		2016–2017		% Change	*P*_trend_
	Mean	95 % CI	Mean	95 % CI	Mean	95 % CI	Mean	95 % CI	Mean	95 % CI		
Men												
All meat (g/d)	300	292, 308	305	297, 314	304	296, 312	299	291, 308	295	287, 303	−2	0·67
Beef (g/d)	133	129, 138	132	127, 137	131	127, 136	127	123, 132	122	117, 127	−9	0·06
Processed meat (g/d)	35	34, 37	34	32, 35	35	34, 37	35	33, 36	33	31, 34	−9	0·43
Poultry (g/d)	48	46, 51	52	50, 55	51	49, 54	53	51, 56	53	50, 55	8	0·04
Fish (g/d)	83	79, 87	87	83, 91	86	82, 90	84	81, 88	88	84, 91	5	0·44
Women												
All meat (g/d)	228	220, 236	233	224, 241	232	223, 240	227	219, 235	223	215, 231	−2	0·17
Beef (g/d)	84	80, 89	83	78, 88	82	78, 87	78	74, 83	73	68, 78	−15	<0·001
Processed meat (g/d)	21	20, 23	19	18, 21	21	19, 23	20	19, 22	19	17, 20	−15	0·07
Poultry (g/d)	43	40, 45	47	44, 49	46	43, 48	48	45, 50	47	45, 50	10	0·05
Fish (g/d)	79	76, 83	83	80, 87	82	78, 86	81	77, 84	84	80, 88	6	0·31

* Mean and 95 % CI are from margins after linear regression with survey period as predictor, adjusted for age and sex. *P* for linear trend across survey years.

### Sociodemographic characteristics of different diet groups

Sociodemographic determinants of dietary choices are presented in Table 4. Compared with omnivores, vegetarians were younger (OR 2·38; 95 % CI 1·01, 5·6) and more likely to be women (OR 1·52; 95 % CI 0·98, 2·35), although this result was non-significant. They were also more likely to have a university degree than omnivores (OR 1·59; 95 % CI 1·01, 2·49). Compared with omnivores, pescatarians were more likely to be women (OR 1·81; 95 % CI 1·10, 3·00) and less likely to be Swiss (OR 0·59; 95 % CI 0·36, 0·98). Flexitarians were more likely to be female (OR 1·57; 95 % CI 1·41, 1·75), more likely to live alone than omnivores (OR 1·49; 95 % CI 1·33, 1·66) and more likely to be Swiss (OR 1·57; 95 % CI 1·38, 1·77). Conversely, individuals with a low income were more likely to follow a vegetarian (OR 1·83; 95 % CI 1·04, 3·21) or flexitarian (OR 1·31; 95 % CI 1·13, 1·53) diet. Smoking status was not associated with dietary regimen.

**Table 4.** Association of diet patterns with sociodemographic and cardiovascular risk factors, Bus Santé study 2005–2017* (*n* 10 797) (Numbers and percentages; odds ratios and 95 % confidence intervals)

	Omnivorous		Flexitarian		Pescatarian		Vegetarian		Flexitarian *v*. omnivorous		Pescatarian *v*. omnivorous		Vegetarian *v*. omnivorous	
	*n*	%	*n*	%	*n*	%	*n*	%	OR	95 % CI	OR	95 % CI	OR	95 % CI
Women	4439	49·5	1020	60·6	42	63·6	50	59·5	1·57	1·41, 1·75	1·81	1·10, 3·00	1·52	0·98, 2·35
Men	4526	50·5	662	39·4	24	36·4	34	40·5	1·00	Ref.	1·00	Ref.	1·00	Ref.
Age categories														
18–44	3708	41·4	670	39·8	27	40·9	45	53·6	0·86	0·73, 1·01	0·60	0·31, 1·15	2·38	1·01, 5·61
45–64	3950	44·1	750	44·6	25	37·9	33	39·3	0·92	0·78, 1·08	0·58	0·30, 1·12	1·79	0·75, 4·27
65+	1307	14·6	262	15·6	14	21·2	6	7·1	1·00	Ref.	1·00	Ref.	1·00	Ref.
Living alone	2852	33·4	680	42·3	26	40·0	38	45·8	1·49	1·33, 1·66	1·35	0·82, 2·23	1·48	0·95, 2·29
Swiss nationality	5536	64·9	1203	74·9	35	53·8	53	63·9	1·57	1·38, 1·77	0·59	0·36, 0·98	1·07	0·68, 1·70
University degree	3878	46·1	773	48·8	35	54·7	49	60·5	1·07	0·96, 1·20	1·34	0·82, 2·20	1·59	1·01, 2·49
Manual occupation	2263	27·9	362	23·7	10	16·1	17	22·4	0·85	0·74, 0·97	0·55	0·28, 1·09	0·80	0·47, 1·38
Household income														
<5000 CHF/month	1663	21·4	374	25·4	17	28·8	24	32·0	1·31	1·13, 1·53	1·47	0·76, 2·83	1·83	1·04, 3·21
5000–9500 CHF/month	3039	39·2	582	39·5	22	37·3	25	33·3	1·10	0·97, 1·26	1·05	0·57, 1·95	1·01	0·58, 1·76
>9500 CHF/month	3057	39·4	518	35·1	20	33·9	26	34·7	1·00	Ref.	1·00	Ref.	1·00	Ref.
Current smoker	1879	22·0	365	22·7	7	10·8	13	15·7	1·06	0·93, 1·21	0·45	0·21, 1·00	0·59	0·33, 1·08
BMI categories														
Normal	4408	51·7	966	61·1	42	65·6	51	72·9	1·00	Ref.	1·00	Ref.	1·00	Ref.
Overweight	2945	34·5	433	27·4	20	31·3	16	22·9	0·72	0·63, 0·82	0·75	0·43, 1·31	0·55	0·31, 0·99
Obese	1172	13·7	183	11·6	2	3·1	3	4·3	0·75	0·63, 0·90	0·18	0·04, 0·75	0·27	0·08, 0·89
Hypercholesterolaemia	3289	42·2	608	40·5	18	29·5	10	13·3	0·95	0·84, 1·08	0·53	0·29, 0·95	0·25	0·12, 0·49
Hypertension	2531	30·4	423	26·8	16	25·0	11	13·4	0·84	0·73, 0·96	0·70	0·38, 1·29	0·45	0·23, 0·89
Diabetes	547	6·9	102	6·7	2	3·3	3	3·8	1·04	0·83, 1·30	0·44	0·11, 1·86	0·75	0·23, 2·42

CHF, Swiss Francs (1 CHF = 1·01 USD as of 14 November 2019).

* OR and 95 % CI from logistic or multinomial logistic regression models with the diet type as predictor, adjusted for age, sex and survey year. Hypertension was defined as having a previous diagnosis or blood pressure ≥140/90 mmHg. Hypercholesterolaemia was defined as having a previous diagnosis or having total blood cholesterol >6·5 mmol/l and HDL <1 mmol/l. Diabetes was defined as self-reported diabetes or a fasting plasma glucose level of ≥7 mmol/l.

### Cardiovascular risk factors

The distribution of cardiovascular risk factor for each dietary regimen is presented in Table 4. Vegetarians were less likely to be overweight (OR 0·55; 95 % CI 0·31, 0·99), obese (OR 0·27; 95 % CI 0·08, 0·89), hypercholesterolaemic (OR 0·25; 95 % CI 0·12, 0·49) and hypertensive (OR 0·45; 95 % CI 0·23, 0·89) when compared with omnivores. Pescatarians were less likely to be obese (OR 0·18; 95 % CI 0·04, 0·75) and less hypercholesterolaemic (OR 0·53; 95 % CI 0·29, 0·95) when compared with omnivores. Finally, flexitarians were generally less likely to be overweight (OR 0·72; 95 % CI 0·63, 0·82), obese (OR 0·75; 95 % CI 0·63, 0·90) and hypertensive (OR 0·84; 95 % CI 0·73, 0·96) than omnivores. Dietary regimen did not influence diabetes status. BMI explained part of the association between flexitarians, vegetarians diets and hypertension as well as between pescatarians and hypercholesterolaemia, so that these associations were no longer significant after accounting for BMI (online Supplementary Table S2). Adjustment for education, occupation and income did not change the significance or the magnitude of our results (online Supplementary Table S3).

Association between cardiovascular risk factors on a continuous scale and dietary choices is presented in Table 5. Vegetarians had a lower BMI −2·22 (95 % CI −3·13, −1·31) kg/m^2^, total cholesterol −0·44 (95 % CI −0·67, −0·21) mmol/l and LDL −0·33 (95 % CI −0·52, −0·13) mmol/l. Pescatarians had a lower BMI at −1·7 (95 % CI −2·72, −0·68) kg/m^2^, a lower total cholesterol at −0·34 (95 % CI −0·60, −0·08) mmol/l and LDL −0·36 (95 % CI −0·58, −0·14) mmol/l. They also had a lower blood pressure with systolic at −4·90 (95 % CI −8·54, −1·27) mmHg and diastolic at −3·31 (95 % CI −5·81, −0·81) mmHg. Flexitarians also had a significant lower BMI at −0·66 (95 % CI −0·88, −0·44) kg/m^2^ and a lower blood pressure with systolic at −0·97 (95 % CI −1·76, −0·17) mmHg and diastolic at −0·66 (95 % CI −1·21, −0·11) mmHg. Adjustment for education, occupation and income did not change the significance or the magnitude of our results (online Supplementary Table S4).

**Table 5.** Association between dietary pattern and biomarkers, Bus Santé study 2005–2017 (*n* 10 797)* (Mean values and standard deviations; coefficients and 95 % confidence intervals)

	*n*	Omnivorous		Flexitarian		Pescatarian		Vegetarian		Flexitarian *v*. omnivorous		Pescatarian *v*. omnivorous		Vegetarian *v*. omnivorous	
		Mean	sd	Mean	sd	Mean	sd	Mean	sd	Coefficient	95 % CI	Coefficient	95 % CI	Coefficient	95 % CI
BMI (kg/m^2^)	10 527	25·3	4·3	24·4	4·3	23·5	3·2	22·5	3·8	−0·66	−0·88, −0·44	−1·70	−2·72, −0·68	−2·22	−3·13, −1·31
Fasting plasma glucose (mmol/l)	10 394	5·2	1·0	5·2	1·1	5	0·5	5	0·9	0·01	**−**0·04, 0·07	−0·25	−0·49, −0·01	**−**0·02	**−**0·23, 0·19
TAG (mmol/l)	10 394	1·3	1·6	1·2	0·8	0·9	0·5	1·1	0·6	**−**0·01	**−**0·09, 0·07	**−**0·34	**−**0·71, 0·02	**−**0·03	**−**0·35, 0·29
Total cholesterol (mmol/l)	10 394	5·4	1·1	5·4	1·1	5	0·9	4·8	1·0	**−**0·01	**−**0·06, 0·05	−0·34	−0·60, −0·08	−0·44	−0·67, −0·21
HDL (mmol/l)	10 394	1·5	0·4	1·6	0·5	1·7	0·5	1·5	0·3	0·03	0·00, 0·05	0·10	**−**0·01, 0·19	**−**0·08	**−**0·16, 0·01
LDL (mmol/l)	10 394	3·4	0·9	3·3	1·0	3	0·9	2·9	0·8	**−**0·03	**−**0·07, 0·02	−0·36	−0·58, −0·14	−0·33	−0·52, −0·13
Systolic blood pressure (mmHg)	10 542	122	17·1	120	17·7	116·2	15·3	114·8	15·3	−0·97	−1·76, −0·17	−4·90	−8·54, −1·27	**−**3·09	**−**6·32, 0·14
Diastolic blood pressure (mmHg)	10 548	73·5	10·9	72·3	11·0	69·4	9·9	69·5	9·8	−0·66	−1·21, −0·11	−3·31	−5·81, −0·81	**−**1·90	**−**4·13, 0·32

* Means and standard deviations are adjusted for age, sex and survey year. Coefficients and 95 % CI are from linear regression and adjusted for age, sex, BMI and survey year.

## Discussion

In this 13 year cross-sectional population-based study, we showed that the prevalence of vegetarians in the Geneva adult population was low and slightly increased from 0·5 to 1·2 % from 2005–2009 to 2016–2017. An additional 1·1 % of the population was pescatarian in 2016–2017, and flexitarians represented 15·6 % of the studied population and remained stable during the study period. Vegans represented less than 0·1 % of the studied population. Compared with omnivores, vegetarians were more likely to be young, have a higher education and a low income; pescatarians were more likely to be women and flexitarians were more likely to be women and had a lower income. Total meat intake remained stable, but both sex reduced their red meat consumption during the studied period with results barely non-significant for men. All dietary regimens excluding/reducing meat intake showed a more favourable cardiovascular profile compared with omnivorous (i.e. lower BMI, lower total cholesterol and hypertension).

The prevalence of vegetarians observed in previous studies varies greatly. Our study found a mean prevalence in vegetarian diet of 0·8 %, which is relatively similar to that reported in a Finnish study^(8)^. The present study was also a cross-sectional examination and used an FFQ to determine the type of diet. The prevalence was slightly lower than that in our study with 0·43 % vegetarians. Others studies have found higher prevalence rates, ranging from 1·7 to 3·9 % in Europe^(6,12,14)^ and in the USA^(13,36)^. Between-study differences in the prevalence of vegetarians could be due to different methods used to identify the type of diet. Indeed, evidence suggests that when people self-report themselves as vegetarians, higher rates are found than when the study design uses a specific questionnaire like the FFQ^(8)^. This could be due to a misunderstanding of the definition of vegetarian, which can have a different meaning for each participant and being influenced by cultural norms or health literacy. In addition, it is likely that the population associates vegetarianism with a positive behaviour and wishes to be associated to it, thus producing an overestimation of its true prevalence^(8)^. For instance, the Swiss Vegetarian association estimates the prevalence of vegetarianism in Switzerland in 2017 to be as high as 14 % (3 % vegans, 11 % vegetarians)^(37)^, but these latter estimates are based on a poll that included self-defined vegetarians rather than on a report on actual food intake as in our study. We also observed a positive trend with the prevalence of the vegetarians having increased from 0·5 % at the beginning of the study to 1·2 % at the end. Several studies mention an increase in vegetarianism, but they only cite surveys conducted by vegetarian associations that may tend to overestimate the prevalence of vegetarians^(2,4,5)^. Two independent population-based studies have assessed trends on the prevalence of vegetarian diets, but they presented limitations in their definition of vegetarians^(6,36)^. One of these focused on self-reported vegetarians for health reasons and showed an increase from 1·6 to 1·9 % between 2002 and 2012 in the USA^(36)^. Our study provides new insight based on an independent data source.

In the present study, vegetarians were younger, had a higher level of education and were more likely to be women compared with omnivores, confirming the findings of previous studies^(8,9)^. The association between being vegetarian and higher level of education could be explained by the fact that individuals having a higher education level may be more health^(38)^ and/or environment^(39)^ conscious. Alternatively, this association may be due to secular trends in education that imply that young individuals have higher educational levels^(17)^. The latter seems less likely since the analyses were age adjusted. Income was inversely associated with the following vegetarian diet, with participants with a higher income being more likely to be omnivores in our study. The link between income and meat consumption seems to vary considerably depending on the country. For instance, a German study found no association between following a vegetarian diet and income^(11)^, whereas a Canadian and French study found an association between low income and the use of a vegetarian diet^(9,18)^. In Switzerland, the price of meat ranks among the most expensive in the world^(40)^. For low-income individuals, meat prices could be a reason to reduce meat consumption^(9)^. Meat consumption is highly dependent on cultural habits and social background, which may explain a variation between countries^(11)^.

Our study found that 15·6 % of the population could be defined as flexitarians. To the best of our knowledge, this is the first population-based study to precisely measure with an FFQ the prevalence of this type of diet. Our prevalence of flexitarians seems relatively similar to another study from the Netherlands^(12)^, which reported a prevalence rate of 11–15 % flexitarians between 2009 and 2011, even though in the present study being flexitarian was self-reported^(12)^. Sociodemographic data on flexitarians are lacking in the literature. Flexitarians were more likely to be female and had a lower income than omnivores. However, unlike vegetarians, they did not have higher education and were not younger than omnivores. Interestingly, flexitarians seem to be a distinct population from vegetarians.

Over the 13 years, the overall total meat intake and processed meat remained stable. However, beef intake decreased by 15 % for women and by 9 % for men, and an increase in poultry consumption was observed. This result is interesting as it suggests that the population may be receptive to public health messages encouraging the reduction of red and processed meat intake. Indeed, red and processed meat intake has been positively associated with higher incidence of CVD, type 2 diabetes, certain cancers^(3,41,42)^ and a higher mortality risk^(41)^.

Compared with omnivores, individuals with reduced meat intake generally showed a more favourable profile in terms of cardiovascular risk factors. All dietary profiles had a lower BMI compared with the omnivores (vegetarians of −2·2 kg/m^2^, pescatarians of −1·7 kg/m^2^ and flexitarian of −0·7 kg/m^2^). Both vegetarians and pescatarians had lower rates of hypercholesterolaemia, and flexitarians had lower values of total cholesterol and LDL. Flexitarians and vegetarians had lower rates of hypertension, and all three diets had lower values of blood pressure compared with omnivores with results barely non-significant for vegetarians, probably due to our small sample size. Adjustment for education, occupation and income did not alter the significance or magnitude of our results. However, as expected, several of the observed associations between diet and cardiovascular risk factors other than BMI seemed to be mediated by BMI, supporting the hypothesis that vegetarian diets may lead to a lower BMI, which could then result in a decrease in cardiovascular risk factors. Overall, our findings confirm the previous results from cross-sectional studies and prospective cohorts^(43–45)^ on the positive link between different vegetarian diets and a positive cardiometabolic profile^(17,20,46–50)^.

It has been proposed that the positive impact on health of vegetarian diets is due to a lower energy density, a lower exposure to harmful components contained in animal food (such as saturated fats, cholesterol, haeme Fe and N-glycolylneuraminic acid) and an increased consumption of protective elements such as fibres and antioxidants^(51)^, along with the positive impact on weight discussed earlier. Although the cross-sectional nature of our study does not allow us to conclude on the causal link between vegetarian diets and cardiovascular risk factors, our results are in line with those from important longitudinal studies and several randomised controlled trials reporting the beneficial effects of a vegetarian diet, with less evidence for flexitarians^(24,43,44,46,48,52,53)^. Finally, we cannot exclude that these described positive effects may be due to unmeasured confounding factors. For example, these beneficial effects may be related to the generally advantaged socio-behavioural profile of vegetarians. In our study, this bias seems to be limited by the fact that our population of vegetarians had a lower income than omnivores, with low income generally being a cardiovascular risk factor^(54)^.

Some limitations to our study need to be acknowledged. The sample of vegetarians was low, which potentially leads to power issues. Moreover, the study design did not allow to account for how long participants had been following a specific diet. Since an individual’s meat consumption can fluctuate from month to month, it is possible that an FFQ conducted over a period of only 4 weeks could lead to a misclassification bias and thus mitigate the overall results. In addition to this, although the Bus Santé study aims to be representative of the Geneva population, recruitment bias cannot be excluded. This could lead to an over- or under-selection of participants with different profiles from the population. However, the prevalence of cardiovascular risk factors found in our study appears to be similar to that found in the general population of Geneva^(55)^.

Our study has several strengths. Unlike other studies where the data came from self-reported online^(9)^ or mailed^(19)^ questionnaires, in our study, the questionnaires, anthropometric measurements and biological data came from standardised measurements performed by trained personnel. Unlike many previous studies^(8,10,11)^, vegetarians were identified through an FFQ rather than a subjective question on the type of diet that participants were following. Evidence has shown that up to 80 % of self-reported vegetarians were in fact omnivores^(8)^. Finally, our results are obtained from a cross-sectional study that was government funded with a recruitment in the general population, independent from parties with a potential conflict of interests such as the industry or vegetarian associations. All these strengths should result in more reliable and objective data.

### Conclusion

In our cross-sectional population study spanning over 13 years, the proportion of vegetarians and pescatarians in the population increased, whereas that of flexitarians remained stable at a remarkably high rate. Vegetarians were younger, had a lower income, higher education and were more likely to be women. Like vegetarians, flexitarians were more prone to be female and had a lower income but contrary to vegetarians they did not have higher education and were not younger than omnivores, suggesting that this population represents a distinct pattern among the different vegetarians diet. Vegetarians, pescatarians and flexitarians had a lower prevalence of cardiovascular risks factors such as high BMI, hypertension or hypercholesterolaemia. Our results confirm previous reports from longitudinal and randomised controlled trials and reiterate that fact that promoting a reduction of meat consumption would not only benefit the planet but also population health.

## Supplementary material

For supplementary material accompanying this paper visit https://doi.org/10.1017/S0007114520001762. click here to view supplementary material
