# Supplementary material for: Vegetarian, pescatarian and flexitarian diets: sociodemographic determinants and association with cardiovascular risk factors in a Swiss urban population
Source: Br J Nutr. 2020 May 18;124(8):844–52. doi: 10.1017/S0007114520001762 (PMC7525113; doi:10.1017/S0007114520001762)
Supplement: Supplementary file 1 [file S0007114520001762sup001.docx]

**Supplementary table 1.** Trends in mean consumption of meat type by gender, Bus Santé study 2005-2017

|  | 2005-09 | 2010-11 | 2012-13 | 2014-15 | 2016-17 |  |  |
| --- | --- | --- | --- | --- | --- | --- | --- |
|  | Mean (95% CI) | Mean (95% CI) | Mean (95% CI) | Mean (95% CI) | Mean (95% CI) | % change | p-trend |
| **Men** |  |  |  |  |  |  |  |
| All meat (g/d) | 293 (284, 303) | 304 (294, 313) | 304 (295, 313) | 296 (287, 304) | 297 (288, 306) | 1 | 0.73 |
| Beef (g/d) | 129 (123, 134) | 131 (126, 136) | 132 (126, 137) | 126 (121, 131) | 123 (118, 128) | -4 | 0.46 |
| Processed meat (g/d) | 34 (32, 36) | 34 (32, 35) | 35 (33, 37) | 35 (33, 37) | 33 (31, 35) | -3 | 0.75 |
| Poultry (g/d) | 48 (45, 51) | 52 (49, 55) | 51 (49, 54) | 51 (49, 54) | 52 (50, 55) | 9 | 0.12 |
| Fish (g/d) | 82 (78, 87) | 87 (83, 91) | 86 (82, 90) | 84 (80, 88) | 88 (84, 92) | 7 | 0.45 |
| **Women** |  |  |  |  |  |  |  |
| All meat (g/d) | 222 (213, 231) | 232 (223, 241) | 233 (224, 242) | 224 (215, 233) | 225 (217, 234) | 2 | 0.57 |
| Beef (g/d) | 81 (75, 86) | 83 (78, 88) | 84 (78, 89) | 78 (73, 83) | 75 (70, 80) | -7 | <0.01 |
| Processed meat (g/d) | 20 (18, 22) | 19 (17, 21) | 21 (19, 23) | 20 (18, 22) | 19 (17, 20) | -5 | 0.26 |
| Poultry (g/d) | 42 (40, 45) | 46 (43, 49) | 46 (43, 48) | 45 (43, 48) | 47 (44, 49) | 10 | 0.15 |
| Fish (g/d) | 79 (75, 83) | 84 (80, 88) | 83 (79, 87) | 80 (76, 84) | 85 (81, 89) | 7 | 0.28 |

Mean and 95% confidence intervals are from margins after linear regression with survey period as predictor, adjusted for age, sex, education, occupation, and income. P for linear trend across survey years.

**Supplementary table 2.** Association of diet patterns with sociodemographic and cardiovascular risk factors, Bus Santé study 2005-2017 (N=10797)

|  | Omnivorous | Flexitarian | Pescatarian | Vegetarian | Flexitarian vs omnivorous | Pescatarian vs omnivorous | Vegetarians vs omnivorous |
| --- | --- | --- | --- | --- | --- | --- | --- |
|  | n (%) | n (%) | n (%) | n (%) | OR (95% CI) | OR (95% CI) | OR (95% CI) |
| Women | 4439 (49.5) | 1020 (60.6) | 42 (63.6) | 50 (59.5) | **1.49 (1.33, 1.66)** | 1.50 (0.90, 2.50) | 1.18 (0.75, 1.85) |
| Men | 4526 (50.5) | 662 (39.4) | 24 (36.4) | 34 (40.5) | 1.00 (ref) | 1.00 (ref) | 1.00 (ref) |
| Age categories |  |  |  |  |  |  |  |
| 18-44 | 3708 (41.4) | 670 (39.8) | 27 (40.9) | 45 (53.6) | **0.80 (0.67, 0.94)** | **0.46 (0.24, 0.90)** | 1.71 (0.72, 4.07) |
| 45-64 | 3950 (44.1) | 750 (44.6) | 25 (37.9) | 33 (39.3) | 0.90 (0.77, 1.05) | 0.53 (0.27, 1.03) | 1.54 (0.64, 3.71) |
| 65+ | 1307 (14.6) | 262 (15.6) | 14 (21.2) | 6 (7.1) | 1.00 (ref) | 1.00 (ref) | 1.00 (ref) |
| Living alone | 2852 (33.4) | 680 (42.3) | 26 (40.0) | 38 (45.8) | **1.45 (1.29, 1.62)** | 1.23 (0.74, 2.04) | 1.30 (0.83, 2.03) |
| Swiss nationality | 5536 (64.9) | 1203 (74.9) | 35 (53.8) | 53 (63.9) | **1.51 (1.33, 1.71)** | **0.53 (0.32, 0.87)** | 0.99 (0.62, 1.57) |
| University degree | 3878 (46.1) | 773 (48.8) | 35 (54.7) | 49 (60.5) | 1.03 (0.92, 1.15) | 1.22 (0.73, 2.01) | 1.30 (0.82, 2.07) |
| Manual occupation | 2263 (27.9) | 362 (23.7) | 10 (16.1) | 17 (22.4) | 0.89 (0.78, 1.02) | 0.57 (0.28, 1.17) | 0.92 (0.52, 1.61) |
| Household income |  |  |  |  |  |  |  |
| <5000 CHF/month | 1663 (21.4) | 374 (25.4) | 17 (28.8) | 24 (32.0) | **1.39 (1.19, 1.62)** | 1.61 (0.82, 3.15) | **1.97 (1.10, 3.54)** |
| 5000-9500 CHF/month | 3039 (39.2) | 582 (39.5) | 22 (37.3) | 25 (33.3) | **1.14 (1.00, 1.30)** | 1.15 (0.62, 2.12) | 1.11 (0.64, 1.95) |
| >9500 CHF/month | 3057 (39.4) | 518 (35.1) | 20 (33.9) | 26 (34.7) | 1.00 (ref) | 1.00 (ref) | 1.00 (ref) |
| Current smoker | 1879 (22.0) | 365 (22.7) | 7 (10.8) | 13 (15.7) | 1.05 (0.92, 1.20) | **0.45 (0.20, 0.99)** | **0.53 (0.28, 0.98)** |
| BMI categories |  |  |  |  |  |  |  |
| Normal | 4408 (51.7) | 966 (61.1) | 42 (65.6) | 51 (72.9) | 1.00 (ref) | 1.00 (ref) | 1.00 (ref) |
| Overweight | 2945 (34.5) | 433 (27.4) | 20 (31.3) | 16 (22.9) | **0.72 (0.63, 0.82)** | 0.75 (0.43, 1.31) | **0.55 (0.31, 0.99)** |
| Obese | 1172 (13.7) | 183 (11.6) | 2 (3.1) | 3 (4.3) | **0.75 (0.63, 0.90)** | **0.18 (0.04, 0.75)** | **0.27 (0.08, 0.89)** |
| Hypercholesterolemia | 3289 (42.2) | 608 (40.5) | 18 (29.5) | 10 (13.3) | 0.98 (0.87, 1.12) | 0.58 (0.32, 1.05) | **0.28 (0.14, 0.55)** |
| Hypertension | 2531 (30.4) | 423 (26.8) | 16 (25.0) | 11 (13.4) | 0.91 (0.79, 1.05) | 0.91 (0.49, 1.69) | 0.61 (0.31, 1.21) |
| Diabetes | 547 (6.9) | 102 (6.7) | 2 (3.3) | 3 (3.8) | 1.16 (0.92, 1.47) | 0.66 (0.16, 2.77) | 0.72 (0.17, 3.00) |

OR, Odds Ratio. BMI, Body Mass Index. CHF, Swiss Francs (1CHF= 1.01USD as of 14.11.2019).

Odds ratios and 95% confidence intervals from logistic or multinomial logistic regression models with diet type as predictor, adjusted for age, sex, body mass index, and survey year.

Hypertension was defined as having a previous diagnosis or blood pressure ≥140/90 mm Hg. Hypercholesterolemia was defined as having a previous diagnosis or having total blood cholesterol >6.5 mmol/L and high density lipoprotein <1 mmol/L. Diabetes was defined as self-reported diabetes or fasting plasma glucose level of ≥7 mmol/L.

**Supplementary table 3.** Association of diet patterns with cardiovascular risk factors, Bus Santé study 2005-2017 (N = 10797)

|  | Omnivorous | Flexitarian | Pescatarian | Vegetarian | Flexitarian vs omnivorous | Pescatarian vs omnivorous | Vegetarians vs omnivorous |
| --- | --- | --- | --- | --- | --- | --- | --- |
|  | n (%) | n (%) | n (%) | n (%) | OR (95% CI) | OR (95% CI) | OR (95% CI) |
| BMI categories |  |  |  |  |  |  |  |
| Normal | 4408 (51.7) | 966 (61.1) | 42 (65.6) | 51 (72.9) | 1.00 (ref) | 1.00 (ref) | 1.00 (ref) |
| Overweight | 2945 (34.5) | 433 (27.4) | 20 (31.3) | 16 (22.9) | **0.69 (0.60, 0.79)** | 0.53 (0.27, 1.04) | **0.47 (0.24, 0.91)** |
| Obese | 1172 (13.7) | 183 (11.6) | 2 (3.1) | 3 (4.3) | **0.73 (0.60, 0.89)** | **0.21 (0.05, 0.90)** | **0.20 (0.05, 0.84)** |
| Hypercholesterolemia | 3289 (42.2) | 608 (40.5) | 18 (29.5) | 10 (13.3) | 0.93 (0.82, 1.06) | **0.47 (0.24, 0.92)** | **0.29 (0.14, 0.59)** |
| Hypertension | 2531 (30.4) | 423 (26.8) | 16 (25.0) | 11 (13.4) | **0.83 (0.72, 0.96)** | 0.58 (0.28, 1.19) | **0.44 (0.22, 0.91)** |
| Diabetes | 547 (6.9) | 102 (6.7) | 2 (3.3) | 3 (3.8) | 1.03 (0.81, 1.31) | 0.52 (0.12, 2.22) | 0.52 (0.12, 2.16) |

OR, Odds Ratio. BMI, Body Mass Index. CHF, Swiss Francs (1CHF= 1.01USD as of 14.11.2019).

Odds ratios and 95% confidence intervals from logistic or multinomial logistic regression models with the diet type as predictor, adjusted for age, sex, survey year, education, occupation, and income.

Hypertension was defined as having a previous diagnosis or blood pressure ≥140/90 mm Hg. Hypercholesterolemia was defined as having a previous diagnosis or having total blood cholesterol >6.5 mmol/L and high density lipoprotein <1 mmol/L. Diabetes was defined as self-reported diabetes or fasting plasma glucose level of ≥7 mmol/L.

**Supplementary table 4.** Association between dietary pattern and biomarkers, Bus Santé study 2005-2017 (N = 10797)

|  |  | Omnivorous | Flexitarian | Pescatarian | Vegetarian | Flexitarian vs omnivorous | Pescatarian vs omnivorous | Vegetarian vs omnivorous |
| --- | --- | --- | --- | --- | --- | --- | --- | --- |
|  | N | mean (SD) | mean (SD) | mean (SD) | mean (SD) | coeff (95% CI) | coeff (95% CI) | coeff (95% CI) |
| Body mass index (kg/m2) | 9378 | 25.3 (4.3) | 24.4 (4.3) | 23.5 (3.2) | 22.5 (3.8) | **-0.62 (-0.85, -0.39)** | **-1.61 (-2.66, -0.56)** | **-2.28 (-3.23, -1.32)** |
| Fasting plasma glucose (mmol/L) | 8973 | 5.2 (1) | 5.2 (1.1) | 5 (0.5) | 5 (0.9) | 0.03 (-0.03, 0.08) | -0.19 (-0.44, 0.05) | -0.04 (-0.27, 0.19) |
| Triglyceride (mmol/L) | 8974 | 1.3 (1.6) | 1.2 (0.8) | 0.9 (0.5) | 1.1 (0.6) | -0.01 (-0.09, 0.08) | -0.35 (-0.73, 0.04) | -0.03 (-0.39, 0.32) |
| Total cholesterol (mmol/L) | 8973 | 5.4 (1.1) | 5.4 (1.1) | 5 (0.9) | 4.8 (1) | -0.01 (-0.06, 0.05) | **-0.38 (-0.65, -0.12)** | **-0.44 (-0.68, -0.20)** |
| HDL (mmol/L) | 8971 | 1.5 (0.4) | 1.6 (0.5) | 1.7 (0.5) | 1.5 (0.3) | **0.02 (0.00, 0.05)** | 0.09 (-0.01, 0.19) | -0.09 (-0.19, 0.00) |
| LDL (mmol/L) | 8966 | 3.4 (0.9) | 3.3 (1) | 3 (0.9) | 2.9 (0.8) | -0.03 (-0.08, 0.03) | **-0.39 (-0.62, -0.17)** | **-0.31 (-0.52, -0.10)** |
| Systolic blood pressure (mm Hg) | 9015 | 122 (17.1) | 120 (17.7) | 116.2 (15.3) | 114.8 (15.3) | **-1.08 (-1.91, -0.25)** | **-5.07 (-8.77, -1.36)** | -2.95 (-6.36, 0.47) |
| Diastolic blood pressure (mm Hg) | 9017 | 73.5 (10.9) | 72.3 (11) | 69.4 (9.9) | 69.5 (9.8) | **-0.74 (-1.31, -0.16)** | **-3.69 (-6.25, -1.13)** | -1.95 (-4.30, 0.41) |

SD, Standard Deviation. HDL, high-density lipoprotein. LDL, low-density lipoprotein

Mean and standard deviation (SD) are adjusted for age, sex, and survey year.

Coefficient and 95% confidence interval are from linear regression, and adjusted for age, sex, survey year, body mass index, education, occupation and income.
